# Understanding brain dysfunction in sepsis

**DOI:** 10.1186/2110-5820-3-15

**Published:** 2013-05-29

**Authors:** Romain Sonneville, Franck Verdonk, Camille Rauturier, Isabelle F Klein, Michel Wolff, Djillali Annane, Fabrice Chretien, Tarek Sharshar

**Affiliations:** 1 Univ Paris Diderot, Sorbonne Paris Cité, Assistance Publique–Hôpitaux de Paris, Hôpital Bichat–Claude-Bernard, Service de Réanimation Médicale et des Maladies Infectieuses, 46, rue Henri-Huchard Cedex 18, Paris 75877, France; 2 Histopathologie Humaine et Modèles Animaux, Département Infection et Epidémiologie, Institut Pasteur, Paris, France; 3 Univ Paris Diderot, Sorbonne Paris Cité, Assistance Publique–Hôpitaux de Paris, Hôpital Bichat–Claude-Bernard, Service de Radiologie, 46, rue Henri-Huchard Cedex 18, Paris 75877, France; 4 Réanimation medico-chirurgicale et EA4342, Hôpital Raymond Poincaré, Université de Versailles Saint-Quentin en Yvelines, Garches, France

**Keywords:** Delirium, Brain dysfunction, Sepsis

## Abstract

Sepsis often is characterized by an acute brain dysfunction, which is associated with increased morbidity and mortality. Its pathophysiology is highly complex, resulting from both inflammatory and noninflammatory processes, which may induce significant alterations in vulnerable areas of the brain. Important mechanisms include excessive microglial activation, impaired cerebral perfusion, blood–brain-barrier dysfunction, and altered neurotransmission. Systemic insults, such as prolonged inflammation, severe hypoxemia, and persistent hyperglycemia also may contribute to aggravate sepsis-induced brain dysfunction or injury. The diagnosis of brain dysfunction in sepsis relies essentially on neurological examination and neurological tests, such as EEG and neuroimaging. A brain MRI should be considered in case of persistent brain dysfunction after control of sepsis and exclusion of major confounding factors. Recent MRI studies suggest that septic shock can be associated with acute cerebrovascular lesions and white matter abnormalities. Currently, the management of brain dysfunction mainly consists of control of sepsis and prevention of all aggravating factors, including metabolic disturbances, drug overdoses, anticholinergic medications, withdrawal syndromes, and Wernicke’s encephalopathy. Modulation of microglial activation, prevention of blood–brain-barrier alterations, and use of antioxidants represent relevant therapeutic targets that may impact significantly on neurologic outcomes. In the future, investigations in patients with sepsis should be undertaken to reduce the duration of brain dysfunction and to study the impact of this reduction on important health outcomes, including functional and cognitive status in survivors.

## Review

Sepsis often is characterized by an early and acute encephalopathy, which is associated with increased morbidity and mortality [1,2]. Patients present with fluctuating mental status changes, inattention, disorganized thinking and therefore match with current criteria for delirium. Delirium is a multifactorial syndrome and several risk factors have been identified during critical illness, including patients’ characteristics (e.g., older age, cognitive impairment), the severity of illness, environmental factors (e.g., sleep deprivation, noisy environment), medications (e.g., benzodiazepines, opioids), and common metabolic disturbances, such as fever, dysnatremias, hypoglycemia, and possibly hyperglycemia [3-5]. Among the myriad of conditions that can induce delirium in critical illness, sepsis, in the form of sepsis-associated encephalopathy (SAE), represents the most frequent and severe cause [6,7]. Diagnosing brain dysfunction in a patient with sepsis implies a systematic diagnostic approach of all potential factors, in addition to sepsis, that can contribute to aggravate or prolong brain dysfunction. The purpose of this review is to provide an overview of brain dysfunction in sepsis focusing on pathophysiology, diagnosis, and potential strategies to improve neurologic outcomes.

## Pathophysiology

### Brain signaling and microglial activation

**Figure 1:**
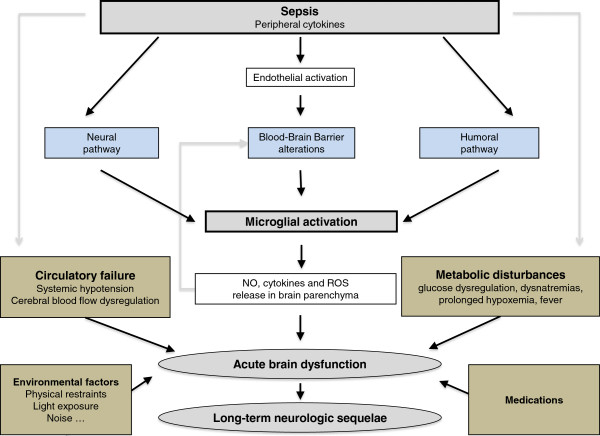
**The response of the brain to systemic infection is physiologically triggered by an activating signal that is mediated by three pathways. **1) The neural pathway that requires activation of primary afferent nerves, such as the vagal or the trigeminal nerves, by involving peripherally produced pathogen-associated molecular patterns (PAMPs) and cytokines. 2) The humoral pathway involves circulating cytokines. They reach the brain at the level of the choroid plexus and the circumventricular organs that lie outside the blood–brain barrier (BBB). 3) The blood–brain barrier alterations induced by the activation of cerebral endothelial cells results in the release of various mediators into the brain. This activation is due to the production, at the early phase of sepsis, of nitric oxide synthase-derived nitric oxide. All of these pathways instigate the activation of microglial cells, which are the resident immune cells of the brain. When activated, microglial cells may negatively affect the brain by the production of nitric oxide, cytokines, and reactive oxygen species that lead to cell death within vulnerable areas of the brain. This production is, in itself, responsible for an increase of the BBB alterations, thus causing a vicious circle of increasing brain dysfunction and injury. These mechanisms are compounded by common metabolic disturbances that occur in septic patients (such as prolonged hyperglycemia, severe hypoxemia), hemodynamic failure, use of medications, and iatrogenic and environmental factors. Septic-associated brain dysfunction may be associated with neurologic sequelae in survivors, including functional and cognitive decline, probably by neurodegenerative and/or ischemic mechanisms.

The encephalopathy in sepsis is considered a diffuse cerebral dysfunction as a consequence of the systemic inflammatory response to an infection, with no direct central nervous system infection (Figure 1). The response to stress is physiologically triggered by an activating signal that is mediated by two pathways. The first one is the vagus nerve, which can detect visceral inflammation through its axonal cytokines receptors: inflammatory products produced in damaged tissues activate afferent signals that are relayed to the nucleus *tractus solitarius* in the brainstem. Subsequent activation of vagus efferent activity inhibits cytokine synthesis in damaged tissues through a cholinergic anti-inflammatory pathway (the inflammatory reflex) [8]. The vagus nerve is also connected to other autonomic nuclei, notably the paraventricular nucleus that controls adrenal axis and vasopressin secretion [9]. The second pathway involves the circumventricular organs (CVOs), which are located near neuroendocrine and neurovegetative nuclei. CVOs are deprived of a blood–brain barrier (BBB) and express components of innate and adaptive immune systems. Once visceral or systemic inflammation is detected by the first or the second pathway, the activating signal will spread to behavioral, neuroendocrine, and neurovegetative centers. Sepsis enhances the transcription of several pro- and anti-inflammatory cytokines and chemokines in the brain, including tumor necrosis factor alpha (TNFα), interleukin-1 beta (IL1β), transforming growth factor beta (TGF β), and monocyte chemoattractant protein 1 (MCP1) [10]. These mediators modulate the expression of α-amino-3-hydroxy-5-methyl-4-isoxazolepropionic acid receptors (AMPARs) and N-methyl-D-aspartate receptors (NMDARs) on neurons, inducing brain dysfunction [11]. Recent studies have suggested the novel importances of IL1β and High Mobility Group Box 1 on the development of cognitive impairment in sepsis survivors [12,13]. These cytokines also modulate NMDARs, with functional consequences on cognition and behavior [14].

Microglial activation may represent one of the earliest changes observed in sepsis-associated encephalopathy and prolonged microglial activation may negatively affect other brain cells [15]. Early microglial activation in sepsis was evidenced in mice models within 4 hours following LPS injection, as assessed by the increased proinflammatory cytokine IL1β level in microglia [16]. Using Positron Emission Tomography (PET) imaging in nonhuman primates, another study demonstrated microglia activation only 1h after LPS-induced systemic inflammation [17]. Moreover, experimental studies suggest that aging may increase the intensity of microglial activation and the production proinflammatory cytokines in the hippocampus, notably IL1β [18,19]. The IL1β-mediated inflammatory process in the hippocampus was confirmed in different models of systemic inflammation, including cecal ligation and puncture and peripheral surgery [20,21]. Numerous experimental studies suggest that these proinflammatory mediators released in the central nervous system at the onset of sepsis will in turn lead to neuronal loss within vulnerable areas of the brain, including the hippocampus [22-24]. Collectively, these findings represent a neuropathological basis for persistent cognitive impairment, hippocampal atrophy, and electroencephalographic changes observed in sepsis survivors [25].

### Endothelial activation and blood–brain barrier dysfunction

Sepsis induces activation of cerebral endothelial cells, which result in BBB dysfunction and release of various mediators into the brain. Experimental data indicate that at the early phase of sepsis, endothelial nitric oxide synthase-derived nitric oxide exhibits proinflammatory characteristics and contributes to the activation and dysfunction of cerebrovascular endothelial cells [26]. The activated endothelium relays the inflammatory response into the brain by releasing proinflammatory cytokines and NO that are able to interact with surrounding brain cells. The other consequences of endothelial activation may include microcirculatory dysfunction, which might compromise cerebral perfusion [27]. Other studies suggest that endotoxemia leads to inflammation in brain, with alteration in BBB, up-regulation of aquaporin 4 (AQP4) and associated edema, neutrophil infiltration, astrocyte activation, as well as apoptotic cell death, all of which appear to be mediated by TNF-alpha signaling through TNF-receptor 1 [28]. Alterations of BBB also have been evidenced in patients with septic shock, with help of brain MRI [29]. BBB breakdown can be localized in the cortex around the Virchow-Robin spaces or have a more diffuse pattern in the whole white matter. It also can predominate in posterior lobes, being consistent with a posterior reversible encephalopathy syndrome [30]. Noninvasive assessment by MRI allowed the identification of new aspects of brain damage in experimental models of sepsis, including cytotoxic and vasogenic edema as well as neuronal damage. These findings highlight the potential applications of MRI techniques for the diagnostic and therapeutic studies in sepsis [31]. Finally, BBB alterations might facilitate the passage of potential neurotoxic factors from the peripheral circulation to the brain. For instance, plasma tryptophan levels are associated with delirium in critically ill patients [32,33]. More recently, increased kynurenine pathway activation, assessed by plasma kynurenine and kynurenine/tryptophan ratio, was found to be associated with fewer days without acute brain dysfunction [33].

Endothelial activation alters vascular tone and induces both microcirculatory dysfunction and coagulopathy, which will in turn favor ischemic and/or hemorrhagic lesions [34]. Neuropathological studies performed in nonsurvivors of septic shock suggest that ischemia is consistently observed in brain areas susceptible to low cerebral flow and that hemorrhages can be found in approximately 10% of cases [34,35]. Furthermore, it has been recently shown that SAE is rather associated with disturbed autoregulation than with altered cerebral blood flow or tissue oxygenation [36].

### Deficit in cholinergic function and alteration of neurotransmission

Deficits in cholinergic function have been postulated to cause delirium and cognitive decline [37]. Global hypocholinergia result from several mechanisms, including impaired acetylcholine synthesis and cholinergic synaptic dysfunction (impairment of presynaptic, synaptic, or postsynaptic functions of acetylcholine). Nicotinic receptors within the brain bind acetylcholine to modulate cognitive functioning, arousal learning, and memory. Use of anesthetic drugs that decrease acetylcholine release (e.g., isoflurane) may impact on cognitive function after surgery [38]. Anticholinergic medications and their metabolites also may induce delirium through competitive antagonism of postsynaptic muscarinic receptors that are more widely distributed throughout the brain. It was recently demonstrated that chronic cholinergic hypoactivity in the basal forebrain represents a major factor of acute brain dysfunction under systemic inflammation [39].

Other significant neurotransmitter alterations have been described during experimental sepsis, involving brain beta-adrenergic, gamma-aminobutyric acid, and serotoninergic pathways [40]. These phenomena seem to predominate in cortex and in hippocampus, and may be mediated by NO, cytokines and prostaglandins [41]. Neurotransmitter balance also is altered by different circulating molecules, such as ammonium, tyrosine, tryptophan, and phenylalanine, whose plasma levels are increased secondary to liver dysfunction and muscle proteolysis [42]. Imbalance between dopaminergic and cholinergic neurotransmission is considered a major mechanism of delirium in criticall*y* ill patients. It also has been hypothesized that reduced cholinergic inhibition of microglia is involved in delirium [15]. However, administration of rivastigmine, a pharmacological agent that may restore cholinergic control of microglia, did not decrease duration of delirium and might have increased mortality of critically ill patients with delirium [43]. Data from studies performed in critically ill patients receiving prolonged mechanical ventilation also suggest that use of GABA-agonists, such as benzodiazepines, is associated with an increased risk of brain dysfunction [44]. Noradrenergic neurotransmission also might be particularly involved in SAE as dexmedetomidine, a selective agonist of alpha2-adrenoceptors expressed in the *locus coeruleus*, is associated with less brain dysfunction and better outcomes in septic patients compared with midazolam [45,46]. These findings were recently confirmed in a recent, multicenter trial, where dexmedetomidine compared with midazolam, improved patient’s ability to communicate [47]. Beneficial effects of dexmedetomidine notably include significant preconditioning and postconditioning effects against ischemic brain injury [48,49]. More recent experimental data suggested that neuroprotective effects of dexmedetomidine against glutamate-induced cell death were mediated by an increase in astrocyte expression of brain-derived neurotrophic factor through an extracellular signal-regulated kinase-dependent pathway [50].

### Oxidative stress, mitochondrial dysfunction, and apoptosis

Experimental data suggest that oxidative damage, assessed by the thiobarbituric acid reactive species and the protein carbonyl assays, occurs early (after 6 hours) in the course of sepsis [51]. Moreover, the combined use of antioxidants (N-acetyl-cysteine and deferoxamine) attenuates oxidative damage in hippocampus 6 hours after sepsis induction [52]. Mitochondrial-mediated apoptosis has been evidenced in experimental sepsis and might be related to a decrease of intracellular anti-apoptotic (bcl-2) and an increase of pro-apoptotic (bax) factors [22]. In patients who had died from septic shock, neuronal and microglial apoptosis have been detected in neurovegetative and neuroendocrine nuclei as well as in amygdala [35]. Intensity of apoptosis correlated with expression of endothelial iNOS. Recent data suggested that Caspase-3 is involved in part in apoptosis in the dentate gyrus cell layer of the hippocampus in septic rats [53]. Additionally to nitric oxide, other pro-apoptotic factors have been incriminated, such as glutamate, TNFα, and hyperglycemia [54].

### Prolonged inflammation and other systemic insults

A recent study performed in critically ill patients suggested that high baseline inflammatory biomarkers of systemic inflammation at admission, such as C-reactive protein and procalcitonin, predicted prolonged periods of acute brain dysfunction, irrespective of whether patients had sepsis or not [55]. In another study performed in 50 patients with systemic inflammatory response syndrome (SIRS), IL-8 was independently associated with delirium [56]. These findings suggest that sustained systemic inflammation may contribute to prolong or aggravate brain dysfunction. Stress hyperglycemia, which commonly develops in critically ill patients, also may aggravate sepsis-induced brain injury. In a recent neuropathological study performed in nonsurvivors of critical illness, hyperglycemia was shown to aggravate critical illness-induced neuropathological changes [57]. Patients with uncontrolled hyperglycemia showed increased microglial activation, an important reduction in the density and activation status of astrocytes, increased neuronal damage, and apoptosis in hippocampus and frontal cortex. Most of these abnormalities were virtually absent with normoglycemia. Interestingly, moderate hyperglycemia was shown to be associated with adverse cognitive outcomes in survivors of the acute respiratory distress syndrome [58]. Prolonged hypoxemia also may contribute to brain dysfunction and injury during sepsis. Recently, in a study performed in survivors of prolonged critical illness, a low partial pressure of arterial oxygen during ICU stay was associated with cognitive and psychiatric impairment at 12 months [59].

### Selective vulnerability

Brain lesions in septic shock have been described in areas of the brain susceptible to ischemia (including Ammon’s horn, the frontal junctional cortex, the lenticular nuclei, the dentate nucleus, and the medullary olive), in the hypothalamic nuclei (supraoptic and paraventricular nuclei), the amygdala, the locus coeruleus, and the medullary autonomic nuclei (nucleus *tractus solitarii*, ambiguous, and parabrachial nuclei) [34,35]. Hippocampal lesions due to inflammatory but also ischemic, hypoxic, or dysglycemic insults [57,60] may explain long-term psychological and cognitive disorders observed in survivors of critical illness [25,61]. Interestingly, reduction of oxidative stress in hippocampus is associated with less cognitive dysfunction in septic rats [52]. There also are arguments for a global brainstem dysfunction during sepsis. First, a recent study suggested that abolition of cough reflex and oculocephalic responses in sedated critically ill patients are associated with death and delirium, respectively [62]. Second, the brainstem nuclei are liable to apoptosis [35] and use of dexmedetomidine, which have antiapoptotic properties [63], is associated with less delirium in septic patients [64]. Finally, impaired sympathetic control of heart rate is frequent and associated with increased mortality in septic patients suggesting a central autonomic regulatory dysfunction [65].

## Diagnosis

### Clinical examination

#### Clinical examination

Detection of acute brain dysfunction in ICU is based on repeated daily neurological examination. Sepsis-associated encephalopathy is characterized by acute changes in mental status, cognition, alteration of sleep/wake cycle, disorientation, impaired attention, and/or disorganized thinking [66]. Sometimes exaggerated motor activity with agitation, and/or hallucinations can be observed and agitation and somnolence can occur alternatively. Other but less frequent motor symptoms include paratonic rigidity, asterixis, tremor and multifocal myoclonus. Physicians have at their disposal validated clinical instruments for detecting brain dysfunction of critically ill patients, including the Confusion Assessment Method for the ICU (CAM-ICU) [6], and the intensive care delirium screening checklist (ICDSC) [67]. Once brain dysfunction is identified, an exhaustive neurological examination assessing neck stiffness, motor responses, muscular strength, plantar and deep tendon reflexes and cranial nerves is mandatory. Although the use of sedative drugs may represent a major limitation in the interpretation of clinical findings, a recent study suggested that assessment of brainstem responses is feasible in sedated critically ill patients and loss of selected responses is predictive of mortality and altered mental status [62]. Assessments of plasma levels of brain injury biomarkers, such as neuron specific enolase and S-100 B-protein, have been proposed for detecting brain dysfunction and injury in sedated patients with sepsis [68,69]. Occurrence of sudden fluctuations in mental status unexplained by modification of sedative infusion rate, occurrence of focal neurological sign, seizure(s) and/or neck stiffness should prompt the physician to consider neuroimaging, EEG and/or lumbar puncture to rule out a direct central nervous system infection.

### Brain imaging

In case of focal neurological signs, a brain computed tomography should be performed to rule out ischemic or hemorrhagic brain injury [29,34]. Brain MRI should be considered systematically in case of persistent encephalopathy after control of sepsis and exclusion of major confounding factors. With use of diffusion-weighted imaging and gradient echo sequences, this tool has a higher sensitivity than CT for detecting acute CNS disorders, such as recent ischemic or hemorrhagic stroke. Several changes have been described in septic patients (Table 1). Moreover, additionally to the importance of etiological diagnosis, assessment of the nature, and extent of brain damage may also influence patient’s treatment.

**Table 1:** Brain MRI patterns in sepsis

**Brain MRI findings**	**References**
*Acute changes*	
Cytotoxic edema (hippocampus, cortex) ischemic lesions	[29,31]
Vasogenic edema	[29,31]
Posterior reversible encephalopathy syndrome (PRES)	[30,70]
*Chronic changes observed in survivors*	
White matter disruption	[71]
Brain atrophy	[25,72]
(frontal cortex, hippocampus)	

### Electroencephalography

In case of seizure(s), including palpebral myoclonus, an EEG will be required to rule out subtle *status epilepticus*. Several electrographic patterns have been described in septic patients (Table 2). Sepsis can be associated with electrographic seizures or periodic epileptiform discharges [73]. In a recent study, generalized periodic discharges were strongly associated with nonconvulsive seizures and nonconvulsive *status epilepticus*[74]. However, the impact of generalized periodic discharges on neurologic outcomes remains to be elucidated. Other EEG abnormalities include increased theta rhythms, triphasic waves and, less often but more pejorative, burst suppression patterns [75].

**Table 2:** Electroencephalographic patterns in sepsis

**Electroencephalographic findings**	**Association with adverse outcome**	**References**
Normal EEG	0	[76]
Theta (mild generalized slowing)	+	[76]
Delta (severe slowing)	+	[76]
Triphasic waves	+	[76]
Periodic epileptiform discharges	+	[73,74,77]
Electrographic seizures	++	[73]
Generalized suppression or burst-suppression	+++	[75,76]

### Causes of encephalopathy requiring specific treatment

Standard laboratory tests should be performed systematically to detect and correct common metabolic disturbances that can cause delirium or coma (such as hypoglycemia, hypercalcemia, hypo- or hypernatremia). In addition to sedatives and analgesics, many classes of drugs currently administered in critically ill patients can induce acute brain dysfunction, notably a number of antibiotics, steroids, and cardiac drugs (Table 3).

**Table 3:** M**edications associated with brain dysfunction in the ICU**

**Agent**	**Mechanism of action**
**Benzodiazepines**	CNS sedation, neuronal inhibition by membrane hyperpolarization (GABA-agonist)
(long- and short-acting)	
**Opioids**	Anticholinergic toxicity, CNS sedation, fecal impaction
**Antibiotics**	Inhibition of GABA-A receptors
Penicillins, cephalosporins, carbapenems, Quinolones	
**Antiarrhythmics**	Strong anticholinergic effects, sodium channel blockage, unknown
Flecaïne, Amiodarone, Digoxin	
**Beta-blockers**	Not yet described, association with delirium
**Diuretics**	Dehydration and electrolyte disturbances
**Steroids**	Anticholinergic toxicity, Increase of catecholamine activity, GABA-agonist, altered serotonin activity
**Inhaled anesthetics**	Beta-amyloïd protein generation, cytotoxicity of beta-amyloïd potentiating, apoptosis-inducing
**Ketamine**	NMDA-antagonism
**Histamine-2 blocking agents**	Anticholinergic toxicity
Cimetidine	
**Nonsteroidal anti-inflammatory drugs**	Blood–brain-barrier permeability
**Anticholinergics**	Anticholinergic toxicity
oxybutynin, bladder antispasmodics	
**Anticonvulsants**	CNS Sedation
phenobarbital, phenytoin	
**Antiparkinsonian agents**	Dopaminergic toxicity
L-Dopa, dopamine agonists, amantadine	
**Antidepressants**	Anticholinergic toxicity
(amitriptyline, imipramine, doxepin)	

*CNS* central nervous system.

Benzodiazepines and opioid withdrawal syndromes may represent an important cause of delirium after discontinuation of sedation. Alcohol withdrawal syndrome often is evoked in a patient with a history of alcohol dependence who develops encephalopathy [78]. The predominance of psychomotor agitation and autonomic signs are suggestive of the diagnosis. The benefit of alcohol withdrawal prophylaxis is unproven. Early and aggressive titration of medication guided by symptoms is the only feature associated with improved outcomes. In malnourished or alcoholic patients, Wernicke’s encephalopathy must always be evoked and treated with intravenous thiamine, especially if there is evidence of ophthalmoplegia or ataxia [79]. Thiamine deficiency can be aggravated by infusion of glucose. Tobacco dependency is a risk factor for delirium in critically ill patients, which may be prevented by use of nicotine patch in chronic smokers [80]. Numerous iatrogenic and/or environmental factors also may aggravate brain dysfunction, such as use of physical restraints, excessive noise, or underexposure to light in the ICU [81,82].

In a patient with unexplained neurologic symptoms (focal neurologic sign or encephalopathy) and bloodstream infection, infective endocarditis should be systematically ruled out as this condition often is associated with neurologic complications [83]. Of note, endocarditis has to be ruled out in the presence of cerebral microbleeds on MRI [84]. Finally, air embolism is an iatrogenic cause of sudden coma, agitation, seizure, or focal neurological signs, and for which hyperbaric oxygen is recommended. It must be emphasized that SAE often is a multifactorial condition. Finally, reappearance or persistence of encephalopathy may indicate that sepsis is not controlled.

## Outcomes

Eidelman et al. showed in a landmark study that approximately one-third of patients with sepsis had a Glasgow coma scale less than 12 and that alteration of alertness and consciousness was an independent prognosis factor, increasing mortality rate up to 63% when Glasgow coma scale drops below 8 [1]. More recent studies have shown that in addition to being highly prevalent in the ICU, delirium is an independent risk factor for threefold increase in mortality, with elderly patients being at an increased risk [7]. Number of days of ICU delirium was associated with higher 1-year mortality after adjustment for relevant covariates in an older ICU population [85]. Mortality also increases with severity of electrophysiological abnormalities, ranging from 0 when EEG is interpreted as normal to 67% when it shows burst suppressions [73,76]. Electrographic seizures and periodic discharges also are associated with increased mortality [29,73-75]. The prognosis value of MRI findings remains to be assessed [29]. The impact of brain dysfunction during sepsis on secondary outcomes is not known but is certainly close to that reported for delirium in critically ill patients, including prolonged length of stay in ICU and hospital, a longer duration of mechanical ventilation and at an extra cost [7,86]. Severe sepsis also is independently associated with substantial and persistent new cognitive impairment and functional disability [87]. A cognitive deficit also has been reported in survivors from the acute respiratory distress syndrome [88-90]. Interestingly, it has been shown that an elevated level of amyloid-beta in intensive care patients with delirium correlates with long-term cognitive impairment [56]. In addition, hippocampal atrophy may represent a determinant of neuropsychological sequelae [25], including depression, anxiety, and posttraumatic stress syndrome [91].

## Therapeutic perspectives

Because there is no specific treatment for SAE yet, treatment should focus on control of infection source and supportive measures, such as management of organ failure(s), prevention of metabolic disturbances, and avoidance of neurotoxic drugs. Preventive strategies to reduce occurrence and duration of brain dysfunction should be applied for every patient admitted to the ICU (Table 4). Symptomatic treatment of delirium and agitation does not differ from that propose in critically ill patients and has been described elsewhere [92]. Adjunctive therapies of septic shock may protect the BBB or reduce endothelial activation, but their effect has not been established. For instance, activated protein C in septic shock patients with impaired consciousness significantly reduced plasma levels of S100-β protein [93]. Steroids have been shown to reduce posttraumatic stress syndrome [94] and prevention of prolonged hyperglycemia also may be neuroprotective [57].

**Table 4:** Potential strategies to reduce brain dysfunction in ICU patients

**Pharmacological measures**		**Type of study**	**References**
	Reduce use of benzodiazepines and opioids	Observational studies	[44,95]
	Perform daily sedation stops	RCT	[96,97]
	Use dexmedetomidine (versus benzodiazepines or propofol) as sedative	RCT	[46,47,98]
	Pain assessment: sedation – analgesia – delirium protocol	Observational studies	[99,100]
	Prevention of metabolic disturbances (severe hypoxemia, fever, dysnatremia(s), prolonged hyperglycemia…)	Observational studies	[4,54,57,59,101]
**Nonpharmacological measures**			
	Sleep protocol	RCT (non-critical care setting)	[102]
	Reorientation and cognitively stimulating activities		
	Rehydration		
	Use of eyeglasses, magnifying lenses, and hearing aids		
	Avoid use of physical restraints	Observational studies	[82,103]
	Early mobilization	RCT	[104]

*RCT* randomized controlled trial.

Various therapeutic interventions have been experimentally tested. Inhibition of iNOS reduces neuronal apoptosis in septic animals but does not improve the state of consciousness and may even aggravate ischemic injuries of the brain [105]. Another study showed that sepsis-induced cognitive impairment at 2 months was prevented in iNOS knockout mice [24]. Experimental studies show a protective effect on the BBB with the use of magnesium [106,107], riluzole [107], hyperbaric oxygen therapy [108], calcium channel blockers, steroids, or anticytokine antibodies [109]. Intravenous immunoglobulins, administered before cecal ligation and perforation, seem to preserve BBB integrity [110]. Regarding oxidative stress, antioxidant treatment with N-acetylcysteine and deferoxamine prevents cognitive impairment in septic mice [52].

## Conclusions

Brain dysfunction is frequent in sepsis but too often neglected, despite its dramatic impact on outcomes. Its pathophysiology is highly complex, resulting from both inflammatory and noninflammatory processes that affect all types of brain cells. The diagnosis of encephalopathy relies essentially on neurological examination, which can lead to specific neurological tests, including EEG and neuroimaging. Brain dysfunction during sepsis is frequently entwined with others factors that have to be screened systematically, including withdrawal syndrome, drugs overdose, and severe metabolic disturbances. Currently, the treatment of sepsis-associated encephalopathy mainly consists of general management of sepsis and prevention of aggravating factors, including metabolic disturbances, drug overdoses, anticholinergic medications, withdrawal syndromes, and Wernicke’s encephalopathy. In the future, investigations should be undertaken to reduce the duration of brain dysfunction and to study the impact of this reduction on important health outcomes, including mortality and functional and cognitive status in survivors. Modulation of microglial activation, prevention of BBB alterations, and use of antioxidants represent relevant therapeutic targets that may impact significantly on neurologic outcomes.

## Competing interests

The authors declare that they have no competing interests.

## Authors’ contributions

RS, FV, CR and TS wrote the manuscript. IFK, MW, DA, FC reviewed the manuscript. All authors read and approved the final manuscript.
